# The Impact of 51 Risk Factors on Life Expectancy in Canada: Findings from a New Risk Prediction Model Based on Data from the Global Burden of Disease Study

**DOI:** 10.3390/ijerph19158958

**Published:** 2022-07-23

**Authors:** Jacek A. Kopec, Eric C. Sayre, Benajir Shams, Linda C. Li, Hui Xie, Lynne M. Feehan, John M. Esdaile

**Affiliations:** 1 School of Population and Public Health, University of British Columbia, Vancouver, BC V6T 1Z3, Canada; 2 Arthritis Research Canada, Vancouver, BC V5Y 3P2, Canada; esayre@arthritisresearch.ca (E.C.S.); lli@arthritisresearch.ca (L.C.L.); hxie@arthritisresearch.ca (H.X.); jesdaile@arthritisresearch.ca (J.M.E.); 3 Fraser Health Authority, Surrey, BC V3T 0H1, Canada; benajirshams86@gmail.com; 4 Department of Physical Therapy, University of British Columbia, Vancouver, BC V6T 1Z3, Canada; lynnefeehan@gmail.com; 5 Faculty of Health Sciences, Simon Fraser University, Burnaby, BC V5A 1S6, Canada; 6 Department of Medicine, University of British Columbia, Vancouver, BC V5Z 1M9, Canada

**Keywords:** life expectancy, risk factors, prediction models, Global Burden of Disease Study

## Abstract

The aims of this study were (1) to develop a comprehensive risk-of-death and life expectancy (LE) model and (2) to provide data on the effects of multiple risk factors on LE. We used data for Canada from the Global Burden of Disease (GBD) Study. To create period life tables for males and females, we obtained age/sex-specific deaths rates for 270 diseases, population distributions for 51 risk factors, and relative risk functions for all disease-exposure pairs. We computed LE gains from eliminating each factor, LE values for different levels of exposure to each factor, and LE gains from simultaneous reductions in multiple risk factors at various ages. If all risk factors were eliminated, LE in Canada would increase by 6.26 years for males and 5.05 for females. The greatest benefit would come from eliminating smoking in males (2.45 years) and high blood pressure in females (1.42 years). For most risk factors, their dose-response relationships with LE were non-linear and depended on the presence of other factors. In individuals with high levels of risk, eliminating or reducing exposure to multiple factors could improve LE by several years, even at a relatively advanced age.

## 1. Introduction

The health impact of risk factors at the individual level is often measured in terms of absolute risk of disease occurrence or mortality over a specified time period. Risk prediction models, such as the Framingham equations [1], have been employed for many years in the prevention of ischemic heart disease. Prediction models and online risk calculators for many other diseases have been developed, e.g., several cancers [2,3], diabetes [4], or osteoporotic fractures [5]. Despite these advances, and a growing emphasis on personalized medicine in the clinical setting [6], personalized prevention based on quantitative risk information is still an exception rather than a norm [7].

A less common but potentially useful alternative to disease-specific risk prediction is to assess the impact of risk factors in terms of all-cause mortality or life expectancy (LE) [8]. Trends in LE and the underlying causes are important for monitoring population health. In the USA and Canada, for example, LE has declined slightly in recent years [9]. The impact of risk factors on LE at the individual level can be assessed by analyzing data from cohort studies in which all-cause mortality is the outcome. Cohort-based LE effects for some risk factors, e.g., smoking [10], physical activity [11], some dietary factors [12], or metabolic factors [13] have been published, but such data are difficult to compare across studies and have not been synthesized in the literature. 

Another approach to assessing the impact of risk factors is to develop a “synthetic” model which utilizes data from many sources to combine information on mortality and relative risks associated with multiple diseases [8]. This approach has been taken by Lim et al. [14] in developing an all-cause mortality calculator using data from the Global Burden of Disease (GBD) Study and other sources [15]. However, a limitation of Lim’s model is that it included only 12 risk factors (body mass index, systolic blood pressure, LDL cholesterol, fasting plasma glucose, seat belt use, smoking, alcohol, physical activity, fruits, vegetables, omega-3 fatty acids, and nuts), whereas GBD generates data for a much larger number of factors. The goal of the current study was to develop a new synthetic life expectancy model based on GBD data, hereafter referred to as the Comprehensive Health and Risk Manager (CHARM), and to estimate LE effects associated with a large number of risk factors. 

## 2. Materials and Methods 

### 2.1. Conceptual Model

CHARM is a computer program that estimates LE and risk of death from specific conditions based on a person-specific risk profile [16]. Model parameters comprise disease-specific death rates by age and sex, age/sex-specific distributions of risk factors, and age/sex-specific relative risk functions for all disease-risk combinations included in the model. These parameters were used to create abridged period life-tables for males and females [17]. For each sex and 5-year age category, the death rate from each disease was obtained as the observed rate for the country of interest multiplied by the overall person-specific relative risk (RR) that depends on the values of all risk factors (risk profile). 

Estimation of LE starts with the specification of the age and sex of the individual, and the values of the risk factors (e.g., systolic blood pressure, smoking, fruit consumption, etc.). For each risk factor, the exposure-specific RR was calculated relative to the country-specific mean exposure for continuous factors and prevalence-weighted relative risk for categorical factors. Dose-response functions included continuous exponential and more flexible interval risk functions for continuous exposures, and ordinal, nominal, and dichotomous functions for categorical exposures [18]. The overall age/sex-specific RR for each disease was calculated under two different models, a model assuming independent effects (to estimate the total effect of each risk factor) and a model accounting for mediation (to estimate the combined effect of multiple factors, including mediators) [19]. In both models, combined effects were computed according to an additive statistical model (an optional multiplicative model is also available) [19]. Cause-specific death rates from all diseases in a given age/sex category were then summed up to obtain the overall death rate, which was used in an abridged period life table to calculate LE [17]. Hence, estimated LE for a male or female with an average level of all factors would be equal to the observed LE for males or females, respectively, in the country of interest. 

To obtain the effect of changes in a risk factor on LE at any age, we developed an (optional) lagged model, which assumes a gradual change in risk following a change in exposure. In this model, we assumed an exponential decay process, with the half-life parameter specific to each exposure-disease pair [20]. Technical details, including mathematical formulas and a description of the computer interface for CHARM, are provided as Supplementary Materials.

### 2.2. Data Sources 

We used data from the 2016 and 2017 GBD studies (GBD 2016 and GBD 2017) to obtain age/sex-specific deaths rates for 270 diseases, including residual categories for comprehensiveness (Table S1, Supplementary Materials), population distributions (or means) for 60 exposures, and relative risk functions for 16,200 disease-exposure pairs. GBD is an international, collaborative research project whose goal is to provide comprehensive and comparable mortality, morbidity and risk factor information for all countries over time [15]. The GBD methodology has been described in detail in numerous publications [21,22,23,24,25,26,27]. Data sources included peer-reviewed scientific publications, government reports, population surveys, administrative databases, vital registration, cancer registries, police reports, sales data, satellite measurements, and other sources. All data sources used in the study are listed in the publicly available Global Health Data Exchange database [28]. Additional details, including GBD data processing methods, are provided as Supplementary Materials.

### 2.3. Model Output

In this study, we used mortality and risk factor exposure data for Canada. We analyzed the effects of 51 out of 60 risk factors available in the model. Five factors that apply to children only and four factors that do not cause death were excluded. We computed LE gains from eliminating each factor, LE values for different levels of exposure to each factor, and provided an example of gains in LE from simultaneous reductions in multiple risk factors at various ages for a person with a complex risk factor profile. 

### 2.4. Model Performance

To assess model performance, we compared LEs for 40 and 60-year-old males and females according to smoking and body mass index (BMI) between our model and the Mortality Population Risk Tool implemented in the Project Big Life calculator developed by Manuel et al. and based on a Canadian cohort (Canadian Community Health Survey) [8,29]. We also compared a 10-year risk of death from cardiovascular disease (CVD) for a 60-year-old male and female between CHARM and the SCORE chart for low-risk countries in Europe [30]. SCORE is a well-established cohort-based CVD risk-of-death calculator [31]. 

## 3. Results

### 3.1. Life Expectancy under Current vs Optimal Distribution of Risk Factors

Life expectancy at birth (LE0) in Canada in 2017 was 79.61 years for males and 83.74 for females. The difference in LE between males and females diminished with age and by age 90, it was less than one year. In Table 1 we show LE0 assuming the optimal level of exposure to each factor and the difference in LE0 between the optimal and current average levels of exposure in the population. 

The greatest difference in LE0 for males was from eliminating smoking (2.45 years), followed by high systolic blood pressure (SBP) (1.51 years), high body mass index (BMI) (1.43 years), high low-density lipoproteins (LDL) cholesterol (0.98 years), and high sodium intake (0.80 years). For females, the greatest difference was for SBP (1.42 years) followed by smoking (1.26), BMI (1.25), LDL (0.84), and sodium (0.68). The effect of eliminating alcohol drinking was 0.21 years for males and −0.02 (slightly detrimental) for females. Increasing physical activity to 4500 MET-min/week would, on average, improve LE0 by 0.17 and 0.21 years for males and females, respectively. Many of the factors studied had a very small impact on LE0 (0.01 years or less), mainly due to relatively low population levels of exposure in Canada, such that the average person (not a person exposed) would not gain much from removing these factors. These include most chemical exposures at work and some environmental factors (Table 1). 

The combined effect of improving all 51 risk factors from the current average to the optimal level (adjusted for mediation) was 6.26 years for males and 5.05 years for females (Table 1), resulting in LE0s of 85.87 years for males and 88.79 for females. The effect of optimizing all dietary factors was 2.19 years in males and 1.84 in females. The impact of prevention can, of course, be much stronger in groups and individuals with higher than average levels of risk. 

### 3.2. LE According to Risk Factor Levels 

For most risk factors, the relationship between exposure and LE0 was non-linear (Table 2, Table 3 and Table 4). For example, for smoking, the effect of one unit of exposure was greater for low levels of smoking, whereas for SBP, BMI, alcohol, and some dietary factors, the opposite was true. Smoking 10 cigarettes a day since age 20 was associated with an LE0 of 78.21 for males and 79.58 for females, compared with 82.06 and 85.00, respectively, for non-smokers (differences of 3.85 and 5.42 years), indicating a stronger impact of smoking on LE0 in females (Table 2). Among the dietary factors, there was a difference of 0.97 years for males and 0.88 years for females when comparing the optimal sodium intake (1 g a day) with a high intake (5.5 g a day). Eating 300 g (about 4 servings) of fruit a day compared to 75 g produced an LE0 difference of 0.66 and 0.55 years for males and females, respectively. For physical activity, the difference in LE0 between 0 and 1125 MET-min per week was 0.46 and 0.39 years for males and females, whereas the same absolute difference between 1125 and 2250 MET-min per week was only 0.13 and 0.12 years, respectively. With respect to alcohol, we considered 0 g/day to be optimal; however, drinking 18 g per day (1 drink is about 14 g) had very little impact on LE0. Drinking 36 g of alcohol per day reduced LE0 by 0.51 and 0.49 years for males and females, respectively.

We evaluated the impact of 7 metabolic factors (Table 3). Of those, a BMI of 35 resulted in an LE0 of 77.56 for males and 81.83 for females, i.e., 3.48 and 3.16 years less than the LE0 for males and females with an optimal BMI of 20. Other metabolic factors with a potentially strong impact on LE0 were systolic blood pressure, LDL cholesterol, blood glucose, and kidney function.

Among the environmental and occupational factors, a high level of exposure to asbestos was associated with a 1.04-year reduction in LE0 in males and 0.85 years in females (Table 4). Work exposure to arsenic, nickel, silica, and particulate matter, gas, and fumes had a relatively strong impact on LE0. Air pollution was a potentially strong factor, with PM_2.5_ levels around 300 μg/m^3^ associated with a reduction in LE0 of more than 2 years. Very high levels of radon exposure also had a significant impact on LE0. 

### 3.3. Effect of Change in Individual Factors and Their Combinations 

In Table 5, Table 6 and Table 7, we illustrate the mediation-adjusted LE impact of changing selected risk factors individually and in combination in a hypothetical person (male and female) with a specified risk profile. For this example, we selected 10 factors that we considered important for population health in Canada, were modifiable, and represented both behavioral and metabolic factors.

In the standard instantaneous (no lag) effect model, conditional LE at a given age following a change in exposure is the same as the LE of a person who has never been exposed. In the lagged model, we assume a gradual change in risk according to an exponential decay process, with a half-life parameter specific to each disease-exposure pair. In the no-lag model, quitting smoking at age 50 in our example (a person who has smoked 10 cigarettes/day since age 20) would produce gains of 3.18 and 4.23 years in males and females, respectively (Table 5). A more realistic effect of quitting would be obtained by assuming that the risk in those who quit will gradually approach the level of risk among lifetime non-smokers, resulting in gains of 2.63 and 3.63 years, as suggested by the lagged model.

The impact of changing the level of a risk factor may depend on the levels of other factors, as shown in Table 6 for the risk profile considered in Table 5. In this example, quitting smoking once other risk factors have been improved produces larger gains in LE50 than those observed previously, i.e., 3.07 years for males and 4.94 for females. For SBP, however, the interaction with other factors is in the opposite direction, such that the impact of reducing SBP is higher when the levels of other risks are also high.

In Table 7, we show the LE impact of modifying 10 risk factors at the same time, at age 30, 50, or 70, for the hypothetical risk profile considered in Table 5. Differences in LE associated with improvements in all 10 factors ranged from 9.51 years in males and 9.64 in females at age 30 to 3.89 and 4.42 years, respectively, at age 70. Figure S1 (Supplementary Materials) illustrates the impact of these changes in risk on survival probability. It shows how, after a change in exposure at a specified age, survival probability departs from the survival curve of persons with a high level of exposure to all 10 factors and gradually approaches the survival curve of those who have never been exposed to such levels of risk.

### 3.4. Comparisons with Other Models

Comparisons of LEs according to the number of cigarettes and BMI between CHARM and the Big Life calculator are shown graphically in Figure S2 (Supplementary Materials). Among male non-smokers, the effect of BMI on LE was slightly lower in our model. Among female non-smokers, effects were similar, but LEs from our model tended to be slightly higher than those from Big Life in younger females and lower in older females (Figure S2C,D). For smoking, LEs from CHARM were higher for males, especially among light smokers, but lower for females, especially among heavy smokers (Figure S2G,H). The mean difference between the models was −0.027 years and the absolute mean difference was 1.96 years. 

In Figure S3 (Supplementary Materials), we compare the risk of dying from CVD over 10 years in the European SCORE charts to the mediation-adjusted risk generated by CHARM for males and females age 60 according to smoking status, SBP and cholesterol level. Using a color scheme similar to the standard SCORE charts, the figure shows a high level of agreement between the two models. Mean absolute differences in predicted risk of dying from CVD over 10 years between the two models ranged from 0.44% for a female smoker to 1.52% for a male smoker. The maximum difference was 3.71% and was observed for a male non-smoker with an SBP of 180 and LDL of 5 mmol/L. 

## 4. Discussion

In this report we described a novel risk and LE model based on GBD data and provided estimates of the LE impact of 51 risk factors. For many of the factors studied, including most occupational and some dietary factors, these are the first published estimates of their impact on LE. When we set all risk factors to their optimal values, life expectancy at birth for Canada increased to 85.87 for males and 88.79 for females. In males, the greatest impact on LE0 could be gained from eliminating smoking, followed by high SBP, high BMI, high LDL cholesterol, and several dietary risks. In females, the greatest impact would be achieved from optimizing SBP, followed by smoking, BMI, LDL, and dietary factors. The effect of changing a given risk factor was often non-linear and depended on the levels of other factors. Reducing exposure to multiple factors resulted in potentially large gains in life expectancy, even at a relatively advanced age. 

### 4.1. Validity of the Conceptual Model

Our model development process has been described briefly in the methods section. A detailed, technical description of the model and the interactive computer program we have developed are given in Supplementary Materials. It should be noted that our LE estimates are not expected to reflect past experience of any particular cohort. Rather, mortality rates used to calculate LE are based on country- and year-specific death rates. For a specific risk profile, the estimated LE should be interpreted as conditional on the assumption that age/sex-specific mortality rates will remain constant (standard assumption for period life tables) [17]. 

Our model generally assumes that risk factors do not interact in their effect on RR, except for mediation effects. We did not incorporate interactions between risk factors because the required data were not available from the GBD and the knowledge base to justify this is not sufficiently robust at this time [32]. However, our model does allow for RRs to vary according to age and sex. Furthermore, LE effects of risk factors depend in a complex way on the levels of other factors because of competing risks of death from different diseases. Our preference for the additive model of independence in the current study is supported by both theoretical considerations and empirical observations. From a theoretical perspective, independence defined as a lack of biological synergy or antagonism is consistent with a lack of interaction on the additive scale [19]. Empirically, our results for subjects with multiple risk factors suggest plausible effects on LE under the additive model. Moreover, all RRs that vary by age in the GBD data show a sub-multiplicative relationship with age [18,27]. 

To accommodate mediation effects, we applied two models. In estimating the individual (total) effect of each factor, without specifying other factors, we used a model without mediation. For example, the effect of BMI in Table 3 includes the direct effect as well as the effect mediated by SBP, LDL, fasting plasma glucose, and impaired kidney function. However, in estimating the combined effect of multiple factors (including mediators), we used the mediation-adjusted model and assumed that all other factors are kept at their specified or (if not specified) mean levels. For example, the BMI effect in Table 5 is the direct effect only and, therefore, is substantially smaller than the total effect. 

### 4.2. Comparisons with Published Results

Our results can be compared with published LE effects of selected exposures from cohort-based models. In one of the first studies of the impact of smoking [33], LE of heavy smokers was 6–7 years lower than that of non-smokers. In subsequent studies, a range of values have been reported [34,35,36]. Doll et al. reported a 10-year difference in median survival age between heavy smokers and non-smokers in male British doctors [10]. Our results are consistent with data from cohort studies, except that the effect of smoking on LE in CHARM is stronger in females compared with males. A possible reason for the failure of cohort-based models to show this effect is a lack of adjustment for a lower average number of cigarettes smoked by female smokers compared with male smokers [37]. 

The association of diet with life expectancy and all-cause mortality has been controversial [38]. Implausible claims for the effects of some foods have been published, suggesting, for example, that consumption of nuts may increase life expectancy by several years [12]. Our results are more plausible. According to CHARM, given the current levels of consumption, removing all dietary risks would increase LE0 by about 2 years. However, consuming large amounts (5–10 times the average) of sugar or processed meat would reduce LE0 by more than a year, and 300 g of fruit a day (compared to none) would improve LE0 by close to a year. A similar effect would be expected from a very high intake of sodium (5–7 g per day), compared with the optimal intake. Given the well-known association between sodium and blood pressure, this estimate seems realistic and agrees with the literature [39]. 

Previous research on physical activity (PA) found a strong impact on all-cause mortality, but few studies provided data on LE [11,40]. Lim et al. reported LE gains of 1.27 years for males and 1.39 for females from eliminating low PA in the USA [14]. In our model, the effect of PA was smaller and was most pronounced at low levels of activity. It is possible that CHARM, which is based on a meta-analysis of published, confounder-adjusted associations of PA with 5 diseases (IHD, stroke, diabetes, colon cancer, and breast cancer) rather than all-cause mortality, underestimates the impact of PA if a larger number of diseases are associated with PA. However, randomized trials thus far do not support a strong association of PA with all-cause mortality [41].

### 4.3. Comparisons with Other Models

Additional empirical evidence of CHARM’s validity is provided in Figures S2 and S3 (Supplementary Materials). These data show that the results from CHARM with respect to the effect of body mass index and smoking on LE broadly agree with those from the Big Life calculator. When discrepancies do occur, the results from CHARM seem equally or more plausible. For example, among males, smoking 40 cigarettes vs. none results in an LE40 difference of 7.83 years according to CHARM and approximately 7 years according to Big Life. However, the dose-response curve in Big Life is relatively flat beyond 5 cigarettes a day, whereas CHARM shows a continuous, albeit diminishing, increase in effect for heavier smokers. Furthermore, the Big Life calculator does not show a clear difference in the impact of smoking on LE between males and females, resulting in some discrepancy between the two models for heavy female smokers. 

Differences in the estimates of risk of death from CVD between CHARM and SCORE were generally smaller than differences observed among established cohort-based models [42]. On average, CHARM estimates tended to be slightly higher for non-smokers, especially males with an extremely high SBP, and slightly lower for smokers. These differences could be due to methodological differences between the two models or actual differences in the joint effects of SBP, cholesterol, and smoking on CVD mortality between the GBD data and the European cohorts from which the SCORE model was derived.

### 4.4. Strengths and Limitations 

CHARM is a comprehensive, interactive risk and life expectancy model that provides estimates for a large number of diseases (270) and risk factors (60). Previously, Lim and colleagues developed a multi-disease mortality risk calculator using GBD data, but their model included only 12 risk factors [14]. 

Unlike most published risk calculators based on statistical prediction models developed from cohort studies, CHARM is a synthetic model that uses all available, up-to-date data from a wide range of sources, collected, processed, and analyzed by the GBD. Cohort-based models are rarely fully representative of the underlying geographical population. Furthermore, parameters from a single cohort study are usually considered less reliable and less generalizable than those from a meta-analysis of many studies. Moreover, data from cohort studies are limited to a set of variables measured in those studies (may not include all important risk factors and confounders), may not collect data repeatedly over time, and may not be publicly available, which makes the model less transparent and verifiable. In addition, models developed from a cohort study may be difficult to update if the study has been discontinued or extensive new data collection and analyses are needed. In contrast, the relative risks and dose-response relationships underlying our estimates are publicly available, easily updatable, and based on meta-analyses of epidemiological studies carefully evaluated for potential biases and adjusted for confounding and mediation [18,27]. The model has been shown to provide plausible, internally consistent results that generally agree with published data and other models.

All results presented in this paper are based on mortality and exposure data for Canada. Therefore, our LE estimates are not strictly applicable to other countries. However, since the relative risk functions are considered by the GBD to be the same for all countries, the relative effects on LE should be generalizable to other populations, especially other high-income countries. Caution is required when applying our data to low and middle income countries because the impact of risk factors on mortality may depend on access to health care. Nonetheless, replacing current parameters with a parameter database for any other country would be straightforward.

A limitation of our model is that data for some factors that affect LE were not available. Specifically, we did not have risk functions for social determinants of health, genetic factors, and pre-existing conditions, as well as some environmental factors (e.g., temperature). Although these factors are generally unmodifiable, their inclusion in future versions of the model may improve the accuracy of predictions. A limitation of any model are potential inaccuracies in the data-derived parameters. This applies mainly to relative risk functions, derived from epidemiological literature, and the lag parameters. Some parameters derived from studies elsewhere may not apply to Canada. There may also be inaccuracies in exposure distribution data. Although uncertainty intervals for these parameters are available from the GBD, our model currently does not provide uncertainty intervals for LE or risk estimates. Therefore, similar to data from other published models, our estimates should be regarded as approximations and treated with caution. Finally, while LE is an important indicator of population health, a measure that reflects both quantity and quality of life, such as health-adjusted life expectancy (HALE) [26], would also be informative and could be considered in future studies.

### 4.5. Practical Applications

Prior studies of the impact of risk calculators on the uptake of preventive measures by individuals have produced mixed results [7,43,44,45]. This may be due to the kind of information the calculator is providing and the way the results are presented [7,45]. When considering preventive interventions at the individual level, the results presented in this report can be useful. For example, data in Table 2, Table 3 and Table 4 can be used to determine which factors are most important to consider for a given patient and to discuss the potential impact and feasibility of risk factor modifications. However, as our data show, the impact of an intervention on LE depends on the risk factor level, the amount of change, patient age, and other, coexisting risks. Usually, there are many realistic combinations of risk factor modifications that result in similar LE gains. A change in a strong factor may accomplish the same result as multiple changes in weaker factors. Therefore, the objective should be to find the intervention that is most effective and suitable given the patient’s risk profile, preferences and goals. To this end, the best approach may be to use our model interactively and compare LE estimates for several changes in the risk profile. For example, for a female with a risk profile considered in Table 3, reducing smoking by 2 cigarettes a day (from 10 to 8), reducing BMI by 5 units (35 to 30), or reducing SBP by 13 mm Hg (150 to 137) result in a similar gain in LE of about 0.5 years. 

## 5. Conclusions

Personalized prevention has been advocated for many years but despite significant progress in risk prediction for specific diseases, the data and tools needed to support large-scale applications of risk models across all diseases and risk factors have been lacking. In this report we estimated the effects of 51 risk factors on LE, derived from a new model that utilizes GBD data on mortality, risk factor distribution, and relative risk functions for 270 diseases. As such, the model is a synthesis of current epidemiological knowledge in a format suitable for immediate implementation. Limitations of the model notwithstanding, we hope this tool, and the data it generates, can assist patients and their doctors in making difficult choices between different options for risk factor modification.

## Figures and Tables

**Table 1:** Life expectancy at birth (LE0) assuming the optimal level of each factor and difference in LE0 between the optimal and current average exposure for 51 risk factors individually.

Risk Factor (Units)	Life Expectancy at Birth		Difference Relative to Baseline		Optimal Level
	Males	Females	Males	Females	
Baseline (current average exposure)	79.61	83.74	0	0	
Smoking (cigarettes/day since age 20)	82.06	85.00	2.45	1.26	0
Systolic blood pressure (mm Hg)	81.12	85.16	1.51	1.42	110
BMI (kg/m^2^)	81.04	84.99	1.43	1.25	20
Blood low-density lipoprotein(LDL) level (mmol/L)	80.59	84.58	0.98	0.84	0.7
Sodium intake (g/day)	80.41	84.42	0.80	0.68	1.0
Whole grain consumption (g/day)	80.23	84.25	0.62	0.51	150
Nuts and seeds consumption (g/day)	80.11	84.10	0.50	0.36	25
Omega-3 fatty acids intake (mg/day)	80.06	84.03	0.45	0.29	300
Fruit consumption (g/day)	80.04	84.04	0.43	0.30	300
Vegetable consumption (g/day)	79.95	84.01	0.34	0.27	430
Blood glucose (mmol/L)	79.94	84.07	0.33	0.33	4.8
Ambient particulate matter (PM 2.5) concentration (μg/m^3^)	79.92	83.99	0.31	0.25	0
Fiber intake (g/day)	79.85	83.93	0.24	0.19	28
Energy intake from polyunsaturated fatty acids (%)	79.83	83.90	0.22	0.16	13
Alcohol consumption (g/day)	79.82	83.72	0.21	−0.02	0
Energy intake from trans fatty acids (%)	79.79	83.89	0.18	0.15	0
Physical activity (MET-min/week)	79.78	83.95	0.17	0.21	4500
Processed meat consumption (g/day)	79.77	83.82	0.16	0.08	0
Kidney function level	79.71	83.94	0.10	0.20	Cat. 5
Bone lead concentration (µg/g)	79.71	83.81	0.10	0.07	0.02
Calcium intake (g/day)	79.70	83.82	0.09	0.08	1.5
Exposure to second-hand smoking	79.70	84.03	0.09	0.29	No
Asbestos exposure at work	79.67	83.74	0.06	<0.01	No/Low
Legume consumption (g/day)	79.67	83.81	0.06	0.07	70
Milk consumption (g/day)	79.67	83.79	0.06	0.05	520
Ambient ozone concentration (ppb)	79.66	83.78	0.05	0.04	29.1
Red meat consumption (g/day)	79.66	83.76	0.05	0.02	0
Sugar-sweetened beverage consumption (g/day)	79.66	83.76	0.05	0.02	0
Particulate matter, gases, and fumes exposure at work (2 categories)	79.65	83.76	0.04	0.02	No
Silica exposure at work	79.64	83.75	0.03	0.01	No
Water quality (12 categories)	79.62	83.74	0.01	<0.01	Cat. 12
Residential radon gas concentration (Bq/m^3^)	79.62	83.74	0.01	<0.01	10
Diesel engine exhaust exposure at work (3 categories)	79.62	83.74	0.01	<0.01	No
Chewing tobacco use (2 categories)	79.62	83.74	0.01	<0.01	No
Intimate partner violence (2 categories)	79.62	83.74	0.01	<0.01	No
Childhood sexual abuse (2 categories)	79.62	83.74	0.01	<0.01	No
Sanitation facility (3 categories)	79.61	83.74	<0.01	<0.01	Cat. 3
Handwashing facility (2 categories)	79.61	83.74	<0.01	<0.01	Cat. 2
Household use of solid fuels (2 categories)	79.61	83.74	<0.01	<0.01	No
Arsenic exposure at work (3 categories)	79.61	83.74	<0.01	<0.01	No
Benzene exposure at work (3 categories)	79.61	83.74	<0.01	<0.01	No
Beryllium exposure at work (3 categories)	79.61	83.74	<0.01	<0.01	No
Cadmium exposure at work (3 categories)	79.61	83.74	<0.01	<0.01	No
Chromium exposure at work (3 categories)	79.61	83.74	<0.01	<0.01	No
Formaldehyde exposure at work (3 categories)	79.61	83.74	<0.01	<0.01	No
Nickel exposure at work (3 categories)	79.61	83.74	<0.01	<0.01	No
Polycyclic aromatic hydrocarbon exposure at work (3 categories)	79.61	83.74	<0.01	<0.01	No
Sulfuric acid exposure at work (3 categories)	79.61	83.74	<0.01	<0.01	No
Trichloroethylene exposure at work (3 categories)	79.61	83.74	<0.01	<0.01	No
Occupation type for asthmagens exposure (9 categories)	79.61	83.74	<0.01	<0.01	Other
Blood hemoglobin level (g/dL)	79.61	83.74	N/A	<0.01	15
All dietary factors	81.80	85.58	2.19	1.84	Optimal
All factors	85.87	88.79	6.26	5.05	Optimal

LE0 values represent total effects (direct and mediated by other factors), except for all factors combined. Risk factors are ordered according to their effect on LE0, from largest to smallest (in males). Baseline LE0 assumes all risk factors at average values. Exposure categories for categorical risk factors are listed in Table S2 (Supplementary Materials). The optimal level is based on the Theoretical Minimum Risk Exposure Level (TMREL) used in GBD studies [27]. Dietary factors in the model are sodium, whole grain, nuts and seeds, omega-3 fatty acids, fruits, vegetables, fibre, processed meat, red meat, polyunsaturated fatty acids, trans fatty acids, calcium, legumes, milk, and sugar-sweetened beverages.

**Table 2:** Life expectancy at birth for selected levels of 21 behavioral risk factors and differences relative to optimal levels.

Risk Factor Levels	Life Expectancy at Birth		LE Difference Relative to Best Level	
	Males	Females	Males	Females
Smoking (cigarettes/day since age 20)				
0	82.06	85.00	---	---
10	78.21	79.58	3.85	5.42
20	76.68	76.99	5.38	8.01
30	75.34	75.03	6.72	9.97
40	74.29	73.84	7.77	11.16
Physical activity (MET-min/week)				
4500	79.78	83.95	---	---
3375	79.69	83.88	0.09	0.07
2250	79.56	83.77	0.22	0.18
1125	79.43	83.65	0.35	0.30
0	78.97	83.26	0.81	0.69
Alcohol consumption (g/day)				
0	79.82	83.72	---	---
18	79.79	83.75	0.03	−0.03
36	79.31	83.23	0.51	0.49
54	78.57	82.58	1.25	1.14
72	77.60	81.82	2.22	1.90
Sodium intake (g/day)				
1	80.41	84.42	---	---
2.5	80.12	84.15	0.29	0.27
4	79.80	83.86	0.61	0.56
5.5	79.44	83.54	0.97	0.88
7	79.06	83.19	1.35	1.23
Fruit consumption (g/day)				
300	80.04	84.04	---	---
225	79.83	83.87	0.21	0.17
150	79.61	83.69	0.43	0.35
75	79.38	83.49	0.66	0.55
0	79.12	83.28	0.92	0.76
Vegetable consumption (g/day)				
430	79.95	84.01	---	---
322.5	79.8	83.9	0.15	0.11
215	79.65	83.78	0.30	0.23
107.5	79.48	83.66	0.47	0.35
0	79.30	83.53	0.65	0.48
Whole grain consumption (g/day)				
150	80.23	84.25	---	---
112.5	80.06	84.11	0.17	0.14
75	79.87	83.96	0.36	0.29
37.5	79.67	83.80	0.56	0.45
0	79.44	83.63	0.79	0.62
Red meat consumption (g/day)				
0	79.66	83.76	---	---
51.25	79.61	83.73	0.05	0.03
102.5	79.57	83.68	0.09	0.08
153.75	79.52	83.64	0.14	0.12
205	79.46	83.59	0.20	0.17
Processed meat consumption (g/day)				
0	79.77	83.82	---	---
22.5	79.46	83.60	0.31	0.22
45	79.12	83.36	0.65	0.46
67.5	78.74	83.10	1.03	0.72
90	78.31	82.80	1.46	1.02
Sugar-sweetened beverage consumption (g/day)				
0	79.66	83.76	---	---
250	79.42	83.61	0.24	0.15
500	79.16	83.44	0.50	0.32
750	78.86	83.25	0.80	0.51
1000	78.52	83.04	1.14	0.72
Milk consumption (g/day)				
520	79.67	83.79	---	---
390	79.65	83.77	0.02	0.02
260	79.63	83.75	0.04	0.04
130	79.60	83.73	0.07	0.06
0	79.58	83.71	0.09	0.08
Legume consumption (g/day)				
70	79.67	83.81	---	---
52.5	79.59	83.76	0.08	0.05
35	79.52	83.71	0.15	0.10
17.5	79.43	83.65	0.24	0.16
0	79.35	83.60	0.32	0.21
Nuts and seeds consumption (g/day)				
25	80.11	84.10	---	---
18.75	79.98	84.00	0.13	0.10
12.5	79.84	83.90	0.27	0.20
6.25	79.68	83.79	0.43	0.31
0	79.51	83.68	0.60	0.42
Fiber intake (g/day)				
28	79.85	83.93	---	---
21	79.73	83.85	0.12	0.08
14	79.61	83.76	0.24	0.17
7	79.48	83.67	0.37	0.26
0	79.34	83.57	0.51	0.36
Calcium intake (g/day)				
1.5	79.70	83.82	---	---
1.125	79.66	83.79	0.04	0.03
0.75	79.62	83.75	0.08	0.07
0.375	79.57	83.71	0.13	0.11
0	79.51	83.66	0.19	0.16
Omega-3 fatty acids intake (mg/day)				
300	80.06	84.03	---	---
225	79.96	83.97	0.10	0.06
150	79.85	83.89	0.21	0.14
75	79.73	83.82	0.33	0.21
0	79.61	83.74	0.45	0.29
Energy intake from polyunsaturated fatty acids (%)				
13	79.83	83.90	---	---
9.75	79.76	83.84	0.07	0.06
6.5	79.68	83.79	0.15	0.11
3.25	79.60	83.73	0.23	0.17
0	79.52	83.67	0.31	0.23
Energy intake from trans fatty acids (%)				
0	79.79	83.89	---	---
1.05	79.62	83.77	0.17	0.12
2.1	79.42	83.65	0.37	0.24
3.15	79.20	83.51	0.59	0.38
4.2	78.95	83.37	0.84	0.52
Chewing tobacco use				
No	79.62	83.74	---	---
Yes	79.32	83.50	0.30	0.24
Childhood sexual abuse				
No	79.62	83.74	---	---
Yes	79.58	83.73	0.04	0.01
Exposure to intimate partner violence (ever)				
No	79.62	83.74	---	---
Yes	79.60	83.73	0.02	0.01

LE0 values are estimated for specific, selected levels of each factor, assumed to be constant over lifetime, and represent total effects (direct and mediated by other factors). For continuous exposures, the levels (5) are ordered from best to worst and evenly distributed. The best level is based on the Theoretical Minimum Risk Exposure Level (TMREL) in GBD studies [27] and the worst level is chosen based on exposure distribution, data availability, and clinical considerations (see Supplementary Materials). Exposure categories for categorical risk factors are listed in Table S2 in Supplementary Materials.

**Table 3:** Life expectancy at birth for selected levels of 7 metabolic risk factors and differences relative to optimal levels.

Risk Factor Levels	Life Expectancy at Birth		LE Difference Relative to Best Level	
	Males	Females	Males	Females
Body mass index (kg/m^2^)				
20	81.04	84.99	---	---
25	80.16	84.15	0.88	0.84
30	79.04	83.14	2.00	1.85
35	77.56	81.83	3.48	3.16
40	75.46	80.00	5.58	4.99
Systolic blood pressure (mm Hg)				
110	81.12	85.16	---	---
127.5	80.01	84.13	1.11	1.03
145	78.26	82.48	2.86	2.68
162.5	75.22	79.42	5.90	5.74
180	69.31	72.61	11.81	12.55
Blood low-density lipoprotein (LDL) cholesterol (mmol/L)				
0.7	80.59	84.58	---	---
2.025	80.14	84.22	0.45	0.36
3.35	79.52	83.78	1.07	0.80
4.675	78.61	83.20	1.98	1.38
6	77.21	82.42	3.38	2.16
Blood glucose (mmol/L)				
4.8	79.94	84.07	---	---
6.1	79.36	83.56	0.58	0.51
7.4	77.79	81.70	2.15	2.37
8.7	77.03	81.10	2.91	2.97
10	76.12	80.37	3.82	3.70
Blood hemoglobin level (g/dL) *				
15	79.61	83.74	---	---
12.75	79.61	83.74	---	<0.01
10.5	79.61	83.73	---	0.01
8.25	79.61	83.73	---	0.01
6	79.61	83.71	---	0.03
Bone lead concentration (µg/g)				
0.02	79.71	83.81	---	---
0.315	79.71	83.80	<0.01	0.01
0.61	79.71	83.80	<0.01	0.01
0.905	79.70	83.80	0.01	0.01
1.2	79.70	83.80	0.01	0.01
Kidney function level				
Category 5	79.71	83.94	---	---
Category 4	79.32	83.73	0.39	0.21
Category 3	78.97	83.38	0.74	0.56
Category 2	76.75	81.94	2.96	2.00
Category 1	74.78	80.58	4.93	3.36

LE0 values are estimated for specific, selected levels of each factor, assumed to be constant over lifetime, and represent total effects (direct and mediated by other factors). For continuous exposures, the levels (5) are ordered from best to worst and evenly distributed. The best level is based on the Theoretical Minimum Risk Exposure Level (TMREL) in GBD studies [27] and the worst level is chosen based on exposure distribution, data availability, and clinical considerations (see Supplementary Materials). Exposure categories for categorical risk factors are listed in Table S2 in Supplementary Materials. * The effect of low blood hemoglobin is only estimated for women.

**Table 4:** Life expectancy at birth for selected levels of 23 environmental and occupational risk factors and differences relative to optimal levels.

Risk Factor Levels	Life Expectancy at Birth		LE Difference Relative to Best Level	
	Males	Females	Males	Females
Ambient particulate matter (PM_2.5_) concentration (μg/m^3^)				
0	79.92	84.09	---	---
150	78.33	82.35	1.59	1.74
300	77.80	81.80	2.12	2.29
450	77.44	81.43	2.48	2.66
600	77.14	81.12	2.78	2.97
Ambient ozone concentration (ppb)				
29.1	79.66	83.78	---	---
47.825	79.62	83.74	0.04	0.04
66.55	79.57	83.70	0.09	0.08
85.275	79.51	83.65	0.15	0.13
104	79.45	83.59	0.21	0.19
Residential radon gas concentration (Bq/m^3^)				
10	79.62	83.74	---	---
207.5	79.19	83.34	0.43	0.4
405	78.59	82.76	1.03	0.98
602.5	77.75	81.94	1.87	1.80
800	76.59	80.78	3.03	2.96
Exposure to second-hand smoke				
No	79.70	84.03	---	---
Yes	79.03	83.45	0.67	0.58
Asbestos exposure at work				
Low/No	79.67	83.74	---	---
High	78.63	82.89	1.04	0.85
Arsenic exposure at work				
No	79.61	83.74	---	---
Low	78.97	83.12	0.64	0.62
High	78.70	82.87	0.91	0.87
Benzene exposure at work				
No	79.61	83.74	---	---
Low	79.53	83.67	0.08	0.07
High	79.39	83.57	0.22	0.17
Beryllium exposure at work				
No	79.61	83.74	---	---
Low	79.61	83.74	<0.01	<0.01
High	79.46	83.60	0.15	0.14
Cadmium exposure at work				
No	79.61	83.74	---	---
Low	79.61	83.74	<0.01	<0.01
High	79.44	83.58	0.17	0.16
Chromium exposure at work				
No	79.61	83.74	---	---
Low	79.61	83.74	<0.01	<0.01
High	79.45	83.59	0.16	0.15
Diesel engine exhaust exposure at work				
No	79.62	83.74	---	---
Low	79.62	83.74	<0.01	<0.01
High	79.20	83.35	0.42	0.39
Formaldehyde exposure at work				
No	79.61	83.74	---	---
Low	79.61	83.74	<0.01	<0.01
High	79.54	83.68	0.07	0.06
Nickel exposure at work				
No	79.61	83.74	---	---
Low	79.15	83.29	0.46	0.45
High	78.63	82.80	0.98	0.94
Polycyclic aromatic hydrocarbon exposure at work				
No	79.61	83.74	---	---
Low	79.61	83.74	<0.01	<0.01
High	79.34	83.48	0.27	0.26
Silica exposure at work				
No	79.64	83.75	---	---
Low	79.18	83.31	0.46	0.44
High	79.04	83.18	0.60	0.57
Sulfuric acid exposure at work				
No	79.61	83.74	---	---
Low	79.58	83.73	0.03	0.01
High	79.50	83.71	0.11	0.03
Trichloroethylene exposure at work				
No	79.61	83.74	---	---
Low	79.61	83.74	<0.01	<0.01
High	79.58	83.72	0.03	0.02
Particulate matter, gases, and fumes exposure at work				
No	79.65	83.89	---	---
Low	79.47	83.78	0.18	0.11
High	79.13	83.55	0.52	0.34
Occupation type for asthmagens exposure				
Other	79.61	83.74	---	---
Administration	79.61	83.74	<0.01	<0.01
Technical	79.61	83.74	<0.01	<0.01
Sales	79.61	83.73	<0.01	0.01
Agriculture	79.61	83.73	<0.01	0.01
Mining	79.61	83.74	<0.01	0.01
Transport	79.61	83.74	<0.01	<0.01
Manufacturing	79.61	83.74	<0.01	<0.01
Services	79.61	83.74	<0.01	<0.01
Household use of solid fuels causing air pollution				
No	79.61	83.74	---	---
Yes	78.73	82.76	0.88	0.98
Water quality				
Category 12	79.62	83.74	---	---
Category 9	79.56	83.68	0.06	0.06
Category 6	79.34	83.41	0.28	0.33
Category 3	79.31	83.37	0.31	0.37
Category 1	79.23	83.28	0.39	0.46
Sanitation facility				
Category 3	79.61	83.74	---	---
Category 2	79.55	83.66	0.06	0.08
Category 1	79.53	83.63	0.08	0.11
Handwashing facility				
Category 2	79.61	83.74	---	---
Category 1	79.53	83.64	0.08	0.10

LE0 values are estimated for specific, selected levels of each factor, assumed to be constant over lifetime, and represent total effects (direct and mediated by other factors). For continuous exposures, the levels (5) are ordered from best to worst and evenly distributed. The best level is based on the Theoretical Minimum Risk Exposure Level (TMREL) in GBD studies [27] and the worst level is chosen based on exposure distribution, data availability, and clinical considerations (see Supplementary Materials). Exposure categories for categorical risk factors are listed in Table S2 in Supplementary Materials.

**Table 5:** An example of gains in LE50 for males and females 50 years of age with 10 risk factors, resulting from a reduction in exposure to each factor individually, according to a no-lag and lagged model, adjusted for mediation.

Risk Factor	Initial Level	Final Level	Males		Females	
			No Lag	Lagged	No Lag	Lagged
Baseline life expectancy			77.56	77.56	79.31	79.31
Smoking (cig./day since age 20)	10	0	3.18	2.63	4.23	3.63
Systolic blood pressure (mm Hg)	150	120	2.20	1.85	1.31	1.15
Low-density lipoproteins (mmol/L)	5	2	1.73	1.42	0.87	0.77
Body mass index (kg/m^2^)	35	25	1.07	0.86	0.75	0.62
Whole grain (g/day)	10	150	0.70	0.57	0.42	0.36
Fruit (g/day)	75	300	0.63	0.50	0.50	0.40
Processed meat (g/day)	50	0	0.52	0.42	0.24	0.21
Alcohol (g/day)	42	0	0.41	0.30	0.40	0.29
Physical activity (MET-min/week)	600	4000	0.32	0.29	0.22	0.20
Sodium (g/day)	7	1	0.11	0.09	0.09	0.07

The lagged model assumes a gradual change in risk following a change in exposure. LE50, life expectancy at age 50 years conditional on surviving until age 50; MET, metabolic equivalent of task. The risk factors are ordered by impact in males.

**Table 6:** An example of cumulative LE50 gains in males and females 50 years of age, resulting from a reduction in exposure to multiple risk factors, considering the levels of other factors and adjusting for mediation.

Risk Factors Ordered from Strongest to Weakest				Risk Factors Ordered from Weakest to Strongest			
Males		Females		Males		Females	
Risk Factors	Cumulative LE50 Gain	Risk Factors	Cumulative LE50 Gain	Risk Factors	Cumulative LE50 Gain	Risk Factors	Cumulative LE50 Gain
Baseline	0.00	Baseline	0.00	Baseline	0.00	Baseline	0.00
Smoking	2.63	Smoking	3.63	Sodium	0.09	Sodium	0.07
SBP	4.73	SBP	5.22	PA	0.38	PA	0.27
LDL	5.58	LDL	5.79	Alcohol	0.70	Meat	0.47
BMI	6.43	BMI	6.76	Meat	1.13	Alcohol	0.78
Grain	6.61	Fruit	6.99	Fruit	1.60	Grain	1.10
Fruit	6.79	Grain	7.12	Grain	2.04	Fruit	1.44
Meat	7.07	Alcohol	7.63	BMI	2.83	BMI	2.07
Alcohol	7.50	Meat	7.86	LDL	3.72	LDL	2.58
PA	7.72	PA	8.05	SBP	4.78	SBP	3.26
Sodium	7.85	Sodium	8.20	Smoking	7.85	Smoking	8.20

Ordering of risk factors is based on individual LE50 effects (Table 5). The initial and final levels of the risk factors are as follows: Smoking 10 and 0 cig./day since age 20; SBP: 150 and 120 mm Hg; BMI: 35 and 25 kg/m^2^; LDL: 5 and 2 mmol/L; Whole grain: 10 and 150 g/day; Fruit: 75 and 300 g/day; Processed meat: 50 and 0 g/day; PA: 600 and 4000 MET-min/week; Alcohol: 42 and 0 g/day; Sodium: 7 and 1 g/day. Data have been obtained from a lagged model. LE50, life expectancy at age 50; SBP, systolic blood pressure; BMI, body mass index; LDL, low-density lipoproteins; PA, physical activity; MET, metabolic equivalent of task.

**Table 7:** An example of conditional LE and LE gains for males and females resulting from changing the level of exposure to 10 risk factors simultaneously, at different ages, adjusted for mediation.

Age at Change	Males			Females		
	LE without Change	LE with Change	Difference	LE without Change	LE with Change	Difference
30 years	75.81	85.32	9.51	77.91	87.55	9.64
50 years	77.56	85.41	7.85	79.31	87.51	8.20
70 years	82.39	86.28	3.89	83.07	87.49	4.42

The initial and final levels of the risk factors (Table 5) are as follows: Smoking 10 and 0 cig./day since age 20; SBP: 150 and 120 mm Hg; BMI: 35 and 25 kg/m^2^; LDL: 5 and 2 mmol/L; Whole grain: 10 and 150 g/day; Fruit: 75 and 300 g/day; Processed meat: 50 and 0 g/day; PA: 600 and 4000 MET-min/week; Alcohol: 42 and 0 g/day; Sodium: 7 and 1 g/day. Data have been obtained from a lagged model. Conditional LE is LE at a given age, conditional on surviving until that age. LE, life expectancy; SBP, systolic blood pressure; BMI, body mass index; LDL, low-density lipoproteins; PA, physical activity; MET, metabolic equivalent of task.

## Data Availability

GBD data used in this study are available from the Global Health Data Exchange, Institute for Health Metrics and Evaluation. Internet address: http://ghdx.healthdata.org/, accessed on 10 January 2021.

## Supplementary Materials

The following supporting information can be downloaded at: https://www.mdpi.com/article/10.3390/ijerph19158958/s1. Section S1: Sources of data; Section S2: User interface, Section S3: Mathematical description of the model; Table S1: List of diseases included in the model in alphabetical order; Table S2: Exposure categories for categorical risk factors; Figure S1: Survival probability (%) for males (A) and females (B) with specified levels of 10 risk factors, according to age at which all risk factors are improved, adjusted for mediation; Figure S2: Comparison of the effect of smoking and body mass index on life expectancy in males and females 40 and 60 years of age, according to CHARM and the Big Life calculator (non-mediated model); and Figure S3: Comparison of CVD mortality over 10 years based on CHARM and the European SCORE chart for a 60-year-old male and female (mediated model).
