# Supplementary material for: The Impact of 51 Risk Factors on Life Expectancy in Canada: Findings from a New Risk Prediction Model Based on Data from the Global Burden of Disease Study
Source: Int J Environ Res Public Health. 2022 Jul 23;19(15):8958. doi: 10.3390/ijerph19158958 (PMC9332720; doi:10.3390/ijerph19158958)
Supplement: Supplementary file 1 [file ijerph-19-08958-s001.zip › ijerph-1711334-supplementary.pdf]

Supplementary Materials

Contents

Section S1. Sources of data …………………………………………………………………………………………. 2

Section S2. User interface ………………………………………………………………………………………….. 3

Section S3. Mathematical description of the model ……………………………………………………………. 4

Section S4. References ………………………………………………………………………………………….. 9

Section S5. Tables and Figures ………………………………………………………………………………………. 10

Table S1. List of diseases included in the model in alphabetical order ……………………………… 10

Table S2. Exposure categories for categorical risk factors ………………………………………………… 17

Figure S1. Survival probability (%) for males (A) and females (B) with specified levels of 10 risk factors, according to age at which all risk factors are improved, adjusted for mediation ………………………………………………………………………………………….. 19

Figure S2. Comparison of the effect of smoking and body mass index on life expectancy in males and females 40 and 60 years of age, according to CHARM and the Big Life calculator ………………………………………………………………………………………… 20

Figure S3. Comparison of CVD mortality over 10 years based on CHARM and the European SCORE chart for a 60-year-old male and female ………………………………………………….. 21

S1. Sources of data

All key parameters in CHARM have been obtained from the Global Burden of Disease Study (GBD). Data sources for GBD estimates are discussed in GBD publications [1-5]. All data sources by country are listed in the publicly available Global Health Data Exchange database [6]. For Canada, 962 data sources have been listed.

Model parameters include disease- and age/sex-specific mortality rates, relative risk functions, and exposure distributions (means and proportions). Deaths by age, sex, and disease for Canada were obtained from vital registration and, for some diseases, cancer registries, and police reports [1]. Exposure information came primarily from epidemiological literature, national and provincial population surveys, including self-report and physical measures surveys, as well as sales data, satellite measurements, and other sources [5].

These primary data were subjected to multi-step data processing and statistical modelling to obtain the final estimates. Specifically, causes of death were re-distributed to eliminate garbage codes and other coding errors. Cause-specific death rates for most causes were modelled with the GBD cause of death ensemble model, which combines estimates from a range of statistical models to obtain the best out of sample predictive validity [1,2]. Exposure data were modelled using a Bayesian meta-regression tool (DisMod-MR 2.1) and a spatiotemporal Gaussian process regression model [5]. Covariates and bias correction methods were employed to ensure accuracy of the data. Special modelling approaches were developed for some diseases and risk factors to address challenges in data availability and reduce bias [1-5].

Age/sex-specific relative risk functions for all risk-disease pairs were derived from meta-analyses of randomized trials and epidemiological studies [5]. The lag (half-life) parameters needed for estimating LE in the counterfactual (intervention) scenarios (range 0-5 years) were obtained from a GBD publication

[7] or derived from epidemiological literature, expert opinion, and clinical and theoretical considerations.

S2. User interface

The model was programmed as an SQL database with stored procedures, and Active Server Pages (ASP) (i.e., VB Script) web connectivity for online use. ASP serves the content to the end user, and acts as a go-between between end user and the SQL back end. Risk parameters are stored in the database, as are the end user responses for up to K scenarios (K is adjustable). The SQL database computes the life tables for the completed scenarios, treating the first scenario as the base-case scenario for the user. In addition to interacting with end users, we have developed an administrative interface (also programmed in VB Script), allowing researchers to modify risk calculators, e.g. upload new questions and risk parameters, or rename, rearrange or regroup exposure questions on customizable pages to be presented to the end user.

For all risk factors, we set a minimum and/or maximum value of exposure the user is allowed to enter. For many factors, the natural minimum was 0. For some factors, especially beneficial dietary factors such as fruit or vegetables, the maximum value allowed was the Theoretical Minimum Risk Exposure Level (TMREL) used in GBD publications [5]. For harmful factors, maximum value for which we obtained estimates was selected based on the literature and generally reflected the population distribution of exposure and / or availability of data for extremely high levels of exposure. For some risk factors, we applied an internal maximum, beyond which the relative risk was frozen, and an external maximum (which the user may enter) that could be higher. For example, we set the internal (external) maximum values for smoking at 60 (100) cigarettes per day. For BMI we set the internal range at 20-50, but allowed weight up to 500 kg and height up to 2.5 m to be entered. For SBP, the internal and external range was the same (110-182 mm Hg). It should be noted that none of these parameters are hard-coded

and all can easily be modified. We felt these restrictions were necessary to avoid the model producing LE estimates for levels of exposure that are implausible and for which accuracy of the estimates is difficult to ascertain. At the same time, we did not want to exclude persons exposed to extreme levels of some risk factors, as the distributions can be highly skewed, hence allowing for higher value to be entered even while freezing the risk estimates at the upper internal bound.

S3. Mathematical description of the model

Step 1: Entering the values of the risk factors.

Estimation of LE starts with the specification of the age and sex of the individual, and the values of the risk factors (e.g., systolic blood pressure, smoking, fruit consumption, etc.). For some risk factors, exposure units are changed or appropriate exposure values are derived (e.g., body mass index is derived from weight and height, MET-min/week are calculated from hours of light, moderate and vigorous activity, pack-years of smoking are calculated from years of smoking and cigarettes per day). For factors that are irrelevant based on age or sex, or factors whose values are unknown, average values for a given population (Canada in our version of the model) are assumed.

Step 2: Calculating the person-specific dose of exposure.

For continuous exposures, the person-specific dose of each exposure at age x is obtained as a difference between the current specified level of exposure in the individual and the population mean:

$$D_{e,i} = y_{e,i} - y_{e,mean} \qquad (1)$$

Here, $D_{e,i}$ is the individual dose of exposure *e* for individual *i*, $y_{e,i}$ is the level of exposure *e* for individual *i* and $y_{e,mean}$ is the average level of exposure *e* in the population (we used Canadian data in our model). For categorical risk factors, relative risk was calculated based on the distribution of exposure in the population (see below).

Step 3: Calculating person-specific relative risks for each exposure-disease pair.

For continuous exposures, relative risks in the GBD database are given per *y* units of exposure (e.g., 100 g/day). Relative risk *R* per one unit of exposure is obtained as:

$$R = R_y^{(\frac{1}{y})} \quad (2)$$

When exponential risk can be assumed, the person-specific relative risk for disease *d* and exposure *e* in individual *i* is:

$$R_{d,e,i} = R_{d,e}^{D_{e,i}} \quad (3)$$

Here, $R_{d,e,i}$ is the relative risk for exposure *e* and disease *d* for individual *i*, $R_{d,e}$ is the relative risk per 1 unit of exposure for exposure *e* and disease *d*, and $D_{e,i}$ is the dose of exposure *e* in person *i*.

For continuous risk factors for which exponential dose-response cannot be assumed, e.g., smoking, physical activity, or alcohol consumption, we used more flexible interval risk functions. For example, relative risks for physical activity (relative to a reference level) were available for 56 specific values of exposure, from 0 to 33,000 MET-min/week, at 600 unit increments. Relative risks for any exposure relative to population mean were obtained by linear interpolation. That is, if $R_{y,int}$ is the relative risk for level *y* of exposure relative to a reference level (obtained by interpolation), and $R_{mean,int}$ is the relative risk for the population mean level of exposure (by interpolation), then the relative risk for level *y* of exposure relative to population mean is:

$$R'_y = \frac{R_{y,int}}{R_{mean,int}} \quad (4)$$

For categorical variables with *c* categories, person-specific relative risk for individual *i* at level *l* of exposure *e* relative to average exposure was obtained as:

$$R'_l = \frac{R_l}{\sum_{j=1}^{c} p_j \times R_j} \quad (5)$$

In this equation, R'_l is relative risk for level *l* of exposure relative to the population mean, R_l is relative

risk for level *l* of exposure relative to a reference level, and p_j is the proportion of the population at level *j* of exposure.

Step 4. Adjusting for mediation

The effects of some risk factors are considered to be partially or fully mediated by other factors. To avoid double counting when estimating the combined effects of multiple factors that include mediators, the relative risks were adjusted for mediation using mediation factors [5] and assuming independent mediation effects, as follows:

$$R'_{d,e} = (R_{d,e} - 1) \times (1 - m_1) \times (1 - m_2) \times \ldots \times (1 - m_k) + 1 \tag{6}$$

Here, $R'_{d,e}$ is relative risk for disease *d* and exposure *e* adjusted for mediation, $R_{d,e}$ is total relative risk (mediated and non-mediated), m_1 is mediation factor 1 (proportion of total effect mediated by factor 1) and *k* is the number of mediators for exposure *e*. Individual effects for each risk factor were obtained as total effects (not adjusted for mediation).

Step 5. Combining relative risks for each disease

Multiple risk factors for a given disease are combined assuming independent effects. This is justified by the lack of sufficient data to support synergistic or antagonistic relationships between the risk factors. However, relative risks can vary according to age and sex and we allow the user to choose an additive or a multiplicative model of independence between the risk factors.

In the additive model, person-specific overall relative risk $R_{d,i}$ for disease *d* is derived as follows:

$$R'_{d,i} = R_{d,e_1,i} + R_{d,e_2,i} + \cdots + R_{d,e_k,i} - k' + 1 = \sum_{j=1}^{k\prime} R_{d,e_j,i} - k' + 1 \tag{7a}$$

$$R''_{d,i} = \frac{1}{R_{d,e_1,i}} + \frac{1}{R_{d,e_2,i}} + \cdots + \frac{1}{R_{d,e_k,i}} - k'' + 1 = \sum_{j=1}^{k\prime\prime} 1/R_{d,e_j,i} - k'' + 1 \tag{7b}$$

$$R_{d,i} = R'_{d,i}/R''_{d,i} \tag{7c}$$

Here, $R'_{d,i}$ is the person-specific overall relative risk over the *k′* disease *d* - exposure pairs that have relative risk ≥1, while $R''_{d,i}$ is the person-specific overall relative risk over the *k′′* disease *d* - exposure pairs that have relative risk <1.

In the multiplicative model,

$$R_{d,i} = R_{d,e_1,i} \times R_{d,e_2,i} \times \ldots \times R_{d,e_k,i} = \prod_{j=1}^{k} R_{d,e_j,i} \qquad (8)$$

where *k* is the number of exposures.

Step 6. Calculating person-specific, disease-specific death rates

Disease-specific death rates for individual *i* are calculated by multiplying baseline (reference) age/sex-specific death rate by the relative risk:

$$h_{d,i} = h_{d,ref} \times R_{d,i} \qquad (9)$$

Step 7. Calculating overall age-specific death rates (adding up disease-specific death rates for each age)

Because each individual can only die from one disease, overall all-cause death rates (combining death rates for all *n* conditions) for individual *i* are calculated as a sum of disease-specific death rates, under the standard independence assumption for competing risks.

$$h_i = \sum_{j=1}^{n} h_{d_j,i} \qquad (10)$$

Step 8. Converting death rates to probabilities of dying in the age interval x to x+1.

The overall age-sex specific death rates are converted to probabilities of death in age interval [a,b) as:

$$q_x = 1 - e^{-h_{x,i}*(b-a)} \qquad (11)$$

These age-specific probabilities are used in the life table to calculate life expectancy for individual *i*.

Step 9: Calculating relative risk for counterfactual scenarios

In the base-case (factual) scenario, we allow the researcher to choose between two options for the evolution of relative risk with age, referred to as fixed exposure and fixed relative risk models. In the

former, we assume that the level of exposure specified at entry remains unchanged over time. Since the mean exposure level changes with age, this implies that the relative risk (which is calculated relative to the mean) will change as well. In the latter, we assume that the level of exposure will change in such a way that the relative risk remains constant over time.

To assess the effect of an intervention, the user selects the target (post-intervention or counterfactual) value of each exposure. Next, the model calculates the relative risk for the target value of exposure (for each disease-exposure pair) assuming instantaneous change in risk. Finally, to obtain the expected post-intervention relative risks at time (age) t, we assume that the relative risk will gradually approach the target value according to an exponential decay model with a half-life parameter λ (specific to each disease-exposure pair). That is:

$$R_t = R_1 + (R_0 - R_1) \times (\frac{1}{2})^{t/\lambda} \quad \text{if } R_0 > R_1 \qquad (12a)$$

or

$$R_t = R_1 - (R_1 - R_0) \times (\frac{1}{2})^{t/\lambda} \quad \text{otherwise} \qquad (12b)$$

Here, R_t is the relative risk at time t post-intervention under counterfactual scenario, R_1 is the target relative risk, R_0 is the pre-intervention relative risk, and λ is the half-life lag parameter (time to 50% reduction in excess relative risk).

Step 10. Calculating counterfactual LE and LE gain

New life expectancy based on the post-intervention level(s) of exposure(s) is calculated as explained above by inserting the new age-specific probabilities of death into the life table. Life expectancy gain (LEG) is:

$$LEG = LE_{counterfact} - LE_{base} \qquad (13)$$

6. References

1. GBD 2017 Causes of Death Collaborators. Global, regional, and national age-sex-specific mortality for 282 causes of death in 195 countries and territories, 1980-2017: a systematic analysis for the Global Burden of Disease Study 2017. Lancet. 2018;392(10159):1736-1788.

2. GBD 2017 Mortality Collaborators. Global, regional, and national age-sex-specific mortality and life expectancy, 1950-2017: a systematic analysis for the Global Burden of Disease Study 2017. Lancet. 2018 Nov 10;392(10159):1684-1735

3. GBD 2017 Disease and Injury Incidence and Prevalence Collaborators. Global, regional, and national incidence, prevalence, and years lived with disability for 354 diseases and injuries for 195 countries and territories, 1990-2017: a systematic analysis for the Global Burden of Disease Study 2017. Lancet. 2018;392(10159):1789-1858.

4. GBD 2017 DALYs and HALE Collaborators. Global, regional, and national disability-adjusted life-years (DALYs) for 359 diseases and injuries and healthy life expectancy (HALE) for 195 countries and territories, 1990-2017: a systematic analysis for the Global Burden of Disease Study 2017. Lancet. 2018;392(10159):1859-1922.

5. GBD 2017 Risk Factor Collaborators. Global, regional, and national comparative risk assessment of 84 behavioural, environmental and occupational, and metabolic risks or clusters of risks for 195 countries and territories, 1990-2017: a systematic analysis for the Global Burden of Disease Study 2017. Lancet. 2018;392(10159):1923-1994.

6. Global Health Data Exchange – GHDx. Internet address: http://ghdx.healthdata.org/. Accessed on January 10, 2021.

7. Murray CJL (Editor), Alan D. Lopez AD (Editor). Global Burden of Disease: A comprehensive assessment of mortality and disability from diseases, injuries, and risk factors in 1990 and projected to 2020 (Global Burden of Disease and Injury, Vol 1). 1st Edition (August 1, 1996). Harvard School of Public Health on behalf of the World Health Organization and the World Bank.

Tables and Figures

Table S1: List of diseases included in the model in alphabetical order.

1	Acne vulgaris
2	Acute glomerulonephritis
3	Acute hepatitis A
4	Acute hepatitis B
5	Acute hepatitis C
6	Acute hepatitis E
7	Acute lymphoid leukemia
8	Acute myeloid leukemia
9	African trypanosomiasis
10	Age-related and other hearing loss
11	Age-related macular degeneration
12	Alcohol use disorders
13	Alcoholic cardiomyopathy
14	Alopecia areata
15	Alzheimer disease and other dementias
16	Amphetamine use disorders
17	Anorexia nervosa
18	Anxiety disorders
19	Aortic aneurysm
20	Appendicitis
21	Asbestosis
22	Ascariasis
23	Asthma
24	Atopic dermatitis
25	Atrial fibrillation and flutter
26	Attention-deficit/hyperactivity disorder
27	Autism spectrum disorders
28	Benign and in situ cervical and uterine neoplasms
29	Benign and in situ intestinal neoplasms
30	Benign prostatic hyperplasia
31	Bipolar disorder
32	Bladder cancer
33	Brain and nervous system cancer
34	Breast cancer
35	Bulimia nervosa
36	Cannabis use disorders
37	Caries of deciduous teeth
38	Caries of permanent teeth

39 Cataract
40 Cellulitis
41 Cervical cancer
42 Chagas disease
43 Chlamydial infection
44 Chronic kidney disease due to diabetes mellitus 1
45 Chronic kidney disease due to diabetes mellitus 2
46 Chronic kidney disease due to glomerulonephritis
47 Chronic kidney disease due to hypertension
48 Chronic kidney disease due to other causes
49 Chronic lymphoid leukemia
50 Chronic myeloid leukemia
51 Chronic obstructive pulmonary disease
52 Cirrhosis and other chronic liver diseases due to alcohol use
53 Cirrhosis and other chronic liver diseases due to hepatitis B
54 Cirrhosis and other chronic liver diseases due to hepatitis C
55 Cirrhosis and other chronic liver diseases due to other causes
56 Cirrhosis due to NASH
57 Coal workers pneumoconiosis
58 Cocaine use disorders
59 Colon and rectum cancer
60 Conduct disorder
61 Conflict and terrorism
62 Congenital heart anomalies
63 Congenital musculoskeletal and limb anomalies
64 Contact dermatitis
65 Cutaneous and mucocutaneous leishmaniasis
66 Cystic echinococcosis
67 Cysticercosis
68 Decubitus ulcer
69 Dengue
70 Diabetes mellitus type 1
71 Diabetes mellitus type 2
72 Diarrhoeal diseases
73 Dietary iron deficiency
74 Digestive congenital anomalies
75 Diphtheria
76 Down syndrome
77 Drug-susceptible tuberculosis
78 Dysthymia
79 Ebola

80 Ectopic pregnancy
81 Edentulism and severe tooth loss
82 Encephalitis
83 Endocarditis
84 Endocrine, metabolic, blood, and immune disorders
85 Endometriosis
86 Epilepsy
87 Oesophageal cancer
88 Executions and police conflict
89 Extensively drug-resistant tuberculosis
90 Female infertility
91 Food-borne trematodiases
92 Fungal skin diseases
93 G6PD deficiency
94 G6PD trait
95 Gallbladder and biliary diseases
96 Gallbladder and biliary tract cancer
97 Gastritis and duodenitis
98 Gastroesophageal reflux disease
99 Genital herpes
100 Genital prolapse
101 Glaucoma
102 Gonococcal infection
103 Gout
104 Guinea worm disease
105 H influenzae type B meningitis
106 Hemolytic disease and other neonatal jaundice
107 HIV/AIDS - Extensively drug-resistant Tuberculosis
108 HIV/AIDS - Multidrug-resistant Tuberculosis without extensive drug resistance
109 HIV/AIDS resulting in other diseases
110 HIV-AIDS - Drug-susceptible Tuberculosis
111 Hodgkin lymphoma
112 Hookworm disease
113 Hypertensive heart disease
114 Indirect maternal deaths
115 Inflammatory bowel disease
116 Inguinal, femoral, and abdominal hernia
117 Interpersonal violence
118 Interstitial lung disease and pulmonary sarcoidosis
119 Intracerebral hemorrhage
120 Invasive Non-typhoidal Salmonella (iNTS)

121 Iodine deficiency
122 IQ shift
123 Ischemic heart disease
124 Ischemic stroke
125 Kidney cancer
126 Klinefelter syndrome
127 Larynx cancer
128 Late maternal deaths
129 Latent tuberculosis infection
130 Leprosy
131 Lip and oral cavity cancer
132 Liver cancer due to alcohol use
133 Liver cancer due to hepatitis B
134 Liver cancer due to hepatitis C
135 Liver cancer due to nonalcoholic steatohepatitis (NASH)
136 Liver cancer due to other causes
137 Low back pain
138 Lower respiratory infections
139 Lymphatic filariasis
140 Major depressive disorder
141 Malaria
142 Male infertility
143 Malignant skin melanoma
144 Maternal abortion and miscarriage
145 Maternal deaths aggravated by HIV/AIDS
146 Maternal hemorrhage
147 Maternal hypertensive disorders
148 Maternal obstructed labour and uterine rupture
149 Maternal sepsis and other maternal infections
150 Measles
151 Meningococcal infection
152 Mesothelioma
153 Migraine
154 Motor neuron disease
155 Multidrug-resistant tuberculosis without extensive drug resistance
156 Multiple myeloma
157 Multiple sclerosis
158 Myelodysplastic, myeloproliferative, and other hematopoietic neoplasms
159 Myocarditis
160 Nasopharynx cancer
161 Near vision loss

162 Neck pain
163 Neonatal encephalopathy due to birth asphyxia and trauma
164 Neonatal preterm birth complications
165 Neonatal sepsis and other neonatal infections
166 Neural tube defects
167 Non-Hodgkin lymphoma
168 Non-melanoma skin cancer (basal-cell carcinoma)
169 Non-melanoma skin cancer (squamous-cell carcinoma)
170 Non-rheumatic calcific aortic valve disease
171 Non-rheumatic degenerative mitral valve disease
172 Onchocerciasis
173 Opioid use disorders
174 Orofacial clefts
175 Osteoarthritis of the hip
176 Osteoarthritis of the knee
177 Other benign and in situ neoplasms
178 Other cardiomyopathy
179 Other cardiovascular and circulatory diseases
180 Other chromosomal abnormalities
181 Other chronic respiratory diseases
182 Other congenital birth defects
183 Other digestive diseases
184 Other drug use disorders
185 Other gynecological diseases
186 Other hemoglobinopathies and hemolytic anemias
187 Other intestinal infectious diseases
188 Other leukemia
189 Other malignant neoplasms
190 Other maternal disorders
191 Other meningitis
192 Other mental disorders
193 Other musculoskeletal disorders
194 Other neglected tropical diseases
195 Other neonatal disorders
196 Other neurological disorders
197 Other non-rheumatic valve diseases
198 Other nutritional deficiencies
199 Other oral disorders
200 Other pharynx cancer
201 Other pneumoconiosis
202 Other sense organ diseases

203 Other sexually transmitted infections
204 Other skin and subcutaneous diseases
205 Other unspecified infectious diseases
206 Other urinary diseases
207 Other vision loss
208 Otitis media
209 Ovarian cancer
210 Pancreatic cancer
211 Pancreatitis
212 Paralytic ileus and intestinal obstruction
213 Paratyphoid fever
214 Parkinson's disease
215 Peptic ulcer disease
216 Periodontal diseases
217 Peripheral vascular disease
218 Pneumococcal meningitis
219 Polycystic ovarian syndrome
220 Premenstrual syndrome
221 Prostate cancer
222 Protein-energy malnutrition
223 Pruritus
224 Psoriasis
225 Pyoderma
226 Rabies
227 Refraction disorders
228 Rheumatic heart disease
229 Rheumatoid arthritis
230 Scabies
231 Schistosomiasis
232 Schizophrenia
233 Seborrhoeic dermatitis
234 Self-harm
235 Sickle cell disorders
236 Sickle cell trait
237 Silicosis
238 Stomach cancer
239 Subarachnoid hemorrhage
240 Sudden infant death syndrome
241 Syphilis
242 Tension-type headache
243 Testicular cancer

244 Tetanus
245 Thalassemias
246 Thalassemias trait
247 Thyroid cancer
248 Tracheal, bronchus, and lung cancer
249 Trachoma
250 Transport injuries
251 Trichomoniasis
252 Trichuriasis
253 Turner syndrome
254 Typhoid fever
255 Unintentional injuries
256 Upper respiratory infections
257 Urinary tract infections
258 Urogenital congenital anomalies
259 Urolithiasis
260 Urticaria
261 Uterine cancer
262 Uterine fibroids
263 Varicella and herpes zoster
264 Vascular intestinal disorders
265 Viral skin diseases
266 Visceral leishmaniasis
267 Vitamin A deficiency
268 Whooping cough
269 Yellow fever
270 Zika virus

Table S2: Exposure categories for categorical risk factors

Risk factor	Categories
Kidney function level	1. Stage 5 chronic kidney disease: GFR<15 ml/min/1.73m2<br>2. Stage 4 chronic kidney disease: GFR=15-29 ml/min/1.73m2<br>3. Stage 3 chronic kidney disease: GFR=30-59 ml/min/1.73m2<br>4. Albuminuria, GFR≥60 ml/min/1.73m2 with ACR>30 mg/g<br>5. None of the above
Exposure to second-hand smoking	1. Yes<br>2. No
Asbestos exposure at work	1. High<br>2. Low/No
Occupational exposures (other than asbestos and noise)	1. High<br>2. Low<br>3. No
Water quality	1. Unimproved water source and untreated drinking water<br>2. Unimproved water source and chlorine/solar treated drinking water<br>3. Unimproved water source and boiled/filtered drinking water<br>4. Improved water source and untreated drinking water<br>5. Improved water source and chlorine/solar treated drinking water<br>6. Improved water source and boiled/filtered drinking water<br>7. Piped water source and untreated drinking water<br>8. Piped water source and chlorine/solar treated drinking water<br>9. Piped water source and boiled/filtered drinking water<br>10. High quality piped water source and untreated drinking water<br>11. High quality piped water source and chlorine/solar treated drinking water<br>12. High quality piped water source and boiled/filtered drinking water
Chewing tobacco use	1. Yes<br>2. No
Intimate partner violence	1. Yes<br>2. No
Childhood sexual abuse	1. Yes<br>2. No
Sanitation facility	1. Open defecation<br>2. Using pit latrine with slab/pour – flush latrine<br>3. Using sewer connection toilets/flush toilets/septic tank connection toilets
Handwashing facility	1. No access to soap, water and/or handwashing station<br>2. Access to soap, water and/or handwashing station
Household use of solid fuels causing air pollution	1. Yes<br>2. No
Noise exposure (85+ decibels) at work	1. High, >90 dB<br>2. Low, 85-90 dB<br>3. No, <85 dB
Occupation type for asthmagens exposure	1. Administration<br>2. Technical

	3. Sales<br>4. Agriculture<br>5. Mining<br>6. Transport<br>7. Manufacturing<br>8. Services<br>9. Other
Occupation type for ergonomic factors	1. Professional, technical and related workers<br>2. Administrative and managerial workers<br>3. Clerical and related workers<br>4. Sales workers<br>5. Service workers<br>6. Agricultural, husbandry, and forestry workers, fishermen and hunters<br>7. Production and related workers, transport, equipment operators, and<br>8. Labourers<br>9. Background
Bullying victimization in childhood/adolescence	1. Current year<br>2-23. Number of years (1-22)<br>24. 23 years prior/never

Categories are ordered from highest to lowest risk, except occupation type. Occupational exposures include: asbestos, arsenic, benzene, beryllium, cadmium, chromium, diesel engine exhaust, formaldehyde, nickel, polycyclic aromatic hydrocarbon, silica, sulphuric acid, trichloroethylene, particulate matter, gases, and fumes, and noise.
GFR, glomerular filtration rate; ACR, albumin-to-creatinine ratio.

A. Males

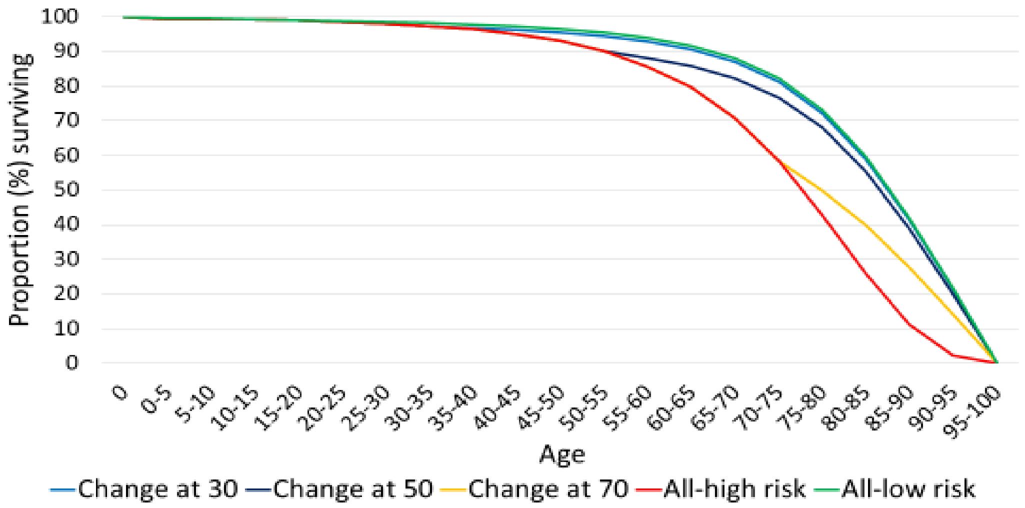

B. Females

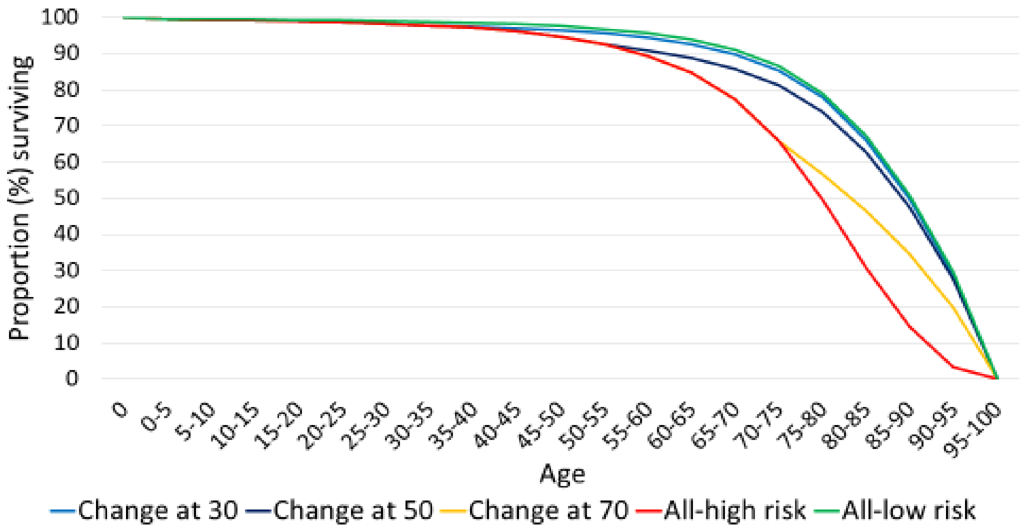

Figure S1. Survival probability (%) for males (A) and females (B) with specified levels of 10 risk factors, according to age at which all risk factors are improved, adjusted for mediation. The initial and final levels of the risk factors are as follows: Smoking (cig./day since age 20): 10 and 0; Systolic blood pressure (mm Hg): 150 and 120; Body mass index (kg/m^2): 35 and 25; Low-density lipoproteins (mmol/L): 5 and 2; Whole grain (g/day): 10 and 150; Fruit (g/day): 75 and 300; Processed meat (g/day): 50 and 0; Physical activity (MET-min/week): 600 and 4,000; Alcohol (g/day): 42 and 0; Sodium (g/day): 7 and 1. All-high risk and All-low risk curves illustrate survival for individuals who do not experience any changes in risk factor levels. All-high risk, all risk factors at the initial (high) level throughout life; All-low risk, all risk factors at the final (low) level throughout life. Data were obtained from a lagged model.

A. Male, age 40, non-smoker

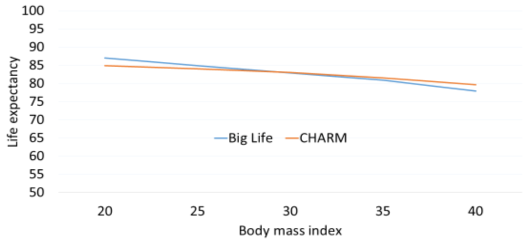

B. Male, age 60, non-smoker

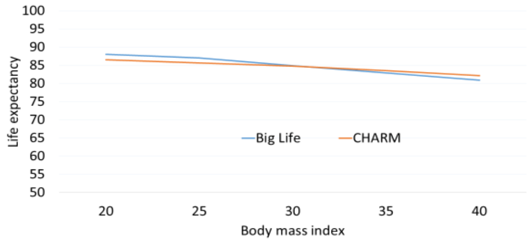

C. Female, age 40, non-smoker

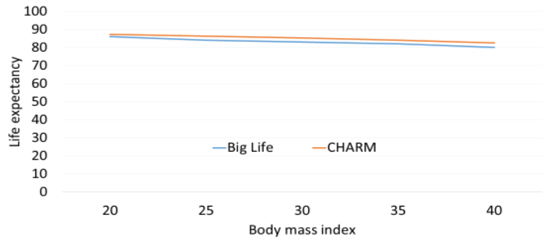

D. Female, age 60, non-smoker

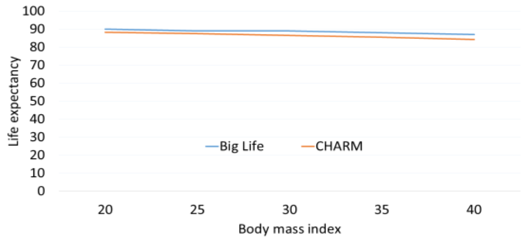

E. Male, age 40

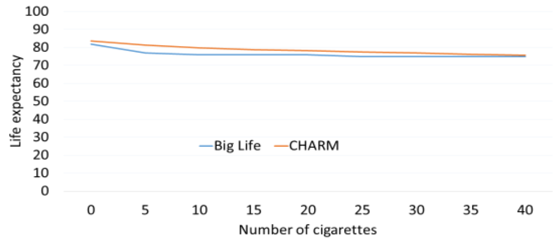

F. Male, age 60

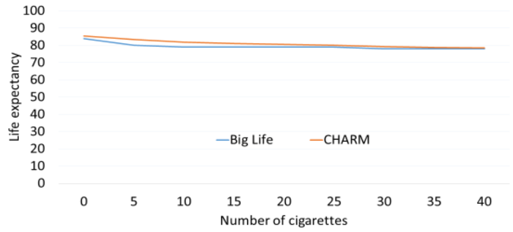

G. Female age 40

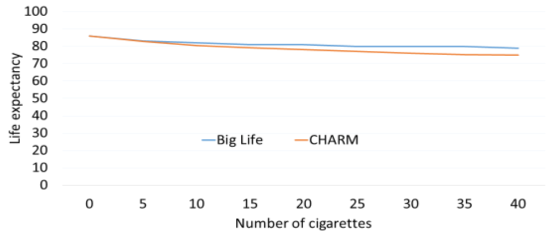

H. Female age 60

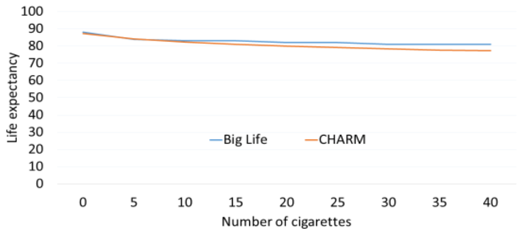

Figure S2. A comparison of the effect of smoking and body mass index on life expectancy in males and females 40 and 60 years of age, according to CHARM and the Big Life calculator (non-mediated model).

A. Female, age 60, non-smoker

SBP	SCORE						CHARM				
180	3	3	3	4	4		4	4	5	5	6
160	2	2	2	2	3		2	2	2	3	3
140	1	1	1	2	2		1	1	1	1	2
120	1	1	1	1	1		1	1	1	1	1
	4	5	6	7	8		2	3	4	5	6
	Total cholesterol (mmol/L)						Low density lipoproteins (mmol/L)				

Mean difference: 0.30%; Mean absolute difference: 0.53%; Maximum difference: 1.72%

B. Female, age 60, smoker

SBP	SCORE						CHARM				
180	5	5	6	7	8		5	5	6	7	8
160	3	4	4	5	5		3	3	4	4	5
140	2	2	3	3	4		2	2	2	3	3
120	1	2	2	2	3		1	1	1	2	2
	4	5	6	7	8		2	3	4	5	6
	Total cholesterol (mmol/L)						Low density lipoproteins (mmol/L)				

Mean difference: -0.38%; Mean absolute difference: 0.44%; Maximum difference: -1.16%

C. Male, age 60, non-smoker

SBP	SCORE						CHARM				
180	5	6	7	8	9		8	9	10	12	12
160	3	4	5	5	6		4	5	5	6	7
140	2	3	3	4	4		2	2	3	3	4
120	2	2	2	3	3		1	1	2	2	2
	4	5	6	7	8		2	3	4	5	6
	Total cholesterol (mmol/L)						Low density lipoproteins (mmol/L)				

Mean difference: 0.72%; Mean absolute difference: 1.22%; Maximum difference: 3.71%

D. Male, age 60, smoker.

SBP	SCORE						CHARM				
180	10	11	13	15	18		9	11	13	15	16
160	7	8	9	11	13		5	6	7	9	10
140	5	5	6	7	9		3	4	5	6	6
120	3	4	4	5	6		2	2	3	3	4
	4	5	6	7	8		2	3	4	5	6
	Total cholesterol (mmol/L)						Low density lipoproteins (mmol/L)				

Mean difference: -1.50%; Mean absolute difference: 1.52%; Maximum difference: -3.44%

Legend: Numbers in colored cells indicate risk (probability) of death from CVD within 10 years.

≥15%	10-14%	5-9%	3-4%	2%	1%	<0.5%

Figure S3. A comparison of CVD mortality over 10 years based on CHARM and the European SCORE chart for a 60-year-old male and female (mediated model). In CHARM, smoker is defined as a person smoking on average 18 (male) or 15 (female)) cigarettes a day since age 20. Total cholesterol in SCORE was replaced with low-density lipoproteins (LDL) in CHARM (assuming *a priori* that LDL represents 50-75% of total cholesterol). Cardiovascular disease in CHARM was defined as a sum of ischemic heart disease, ischemic stroke, intracerebral hemorrhage, hypertensive heart disease, and other cardiovascular disease.
